# Genetic Recombination and *Cryptosporidium hominis* Virulent Subtype IbA10G2

**DOI:** 10.3201/eid1910.121361

**Published:** 2013-10

**Authors:** Na Li, Lihua Xiao, Vitaliano A. Cama, Ynes Ortega, Robert H. Gilman, Meijin Guo, Yaoyu Feng

**Affiliations:** East China University of Science and Technology, Shanghai, China (N. Li, M. Guo, Y. Feng);; Centers for Disease Control and Prevention, Atlanta, Georgia, USA (N. Li, L. Xiao, V.A. Cama);; University of Georgia, Griffin, Georgia, USA (Y. Ortega);; Johns Hopkins University, Baltimore, Maryland, USA (R.H. Gilman)

**Keywords:** Cryptosporidium hominis, genetic recombination, subtype, virulence, molecular epidemiology, cryptosporidiosis, population genetics, parasites, subtype IbA10G2

## Abstract

Little is known about the emergence and spread of virulent subtypes of *Cryptosporidium hominis*, the predominant species responsible for human cryptosporidiosis. We conducted sequence analyses of 32 genetic loci of 53 *C. hominis* specimens isolated from a longitudinally followed cohort of children living in a small community. We identified by linkage disequilibrium and recombination analyses only limited genetic recombination, which occurred exclusively within the 60-kDa glycoprotein gene subtype IbA10G2, a predominant subtype for outbreaks in industrialized nations and a virulent subtype in the study community. Intensive transmission of virulent subtype IbA10G2 in the study area might have resulted in genetic recombination with other subtypes. Moreover, we identified selection for IbA10G2 at a 129-kb region around the 60-kDa glycoprotein gene in chromosome 6. These findings improve our understanding of the origin and evolution of *C. hominis* subtypes and the spread of virulent subtypes.

*Cryptosporidium* spp. are emerging pathogens of humans and a variety of vertebrates, and cause severe diarrhea in immunocompetent and immunocompromised persons (*1*). *Cryptosporidium hominis* is responsible for >70% of human infections in most areas, especially North America and developing countries (*2*). *C. hominis* is primarily transmitted anthroponotically, has several transmission routes, and causes numerous waterborne outbreaks of diarrheal illness each year in the United States and other industrialized nations.

Among several *C. hominis* subtype groups (e.g., Ia, Ib, Id, Ie, If, Ig) identified by sequence analysis of the 60-kDa glycoprotein (gp60) gene, the Ib subtype is the major subtype responsible for waterborne and foodborne outbreaks of cryptosporidiosis in many countries. Subtype IbA10G2 has been found in ≈50% of *C. hominis–*associated outbreaks in the United States, including the massive outbreak in Milwaukee, Wisconsin, USA, in 1993 (*2*,*3*). It is the only subtype identified in cryptosporidiosis outbreaks by *C. hominis* in countries in Europe and in Australia (*4*–*11*). In a longitudinal birth-cohort study of cryptosporidiosis in a periubran shantytown in Lima, Peru, IbA10G2 was more virulent than other *C. hominis* subtypes (*12*). Genetic determinants for virulence of *Cryptosporidium* spp. and reasons for emergence of virulent subtypes are poorly understood because of availability of only limited genomic sequence data and lack of robust cultivation systems and genetic manipulation tools (*13*).

We conducted a comparative population genetic analysis of virulent *C. homini*s subtype IbA10G2 in children living in a periurban community in Lima, Peru, by multilocus sequence typing (MLST) of 32 genetic markers. Data obtained should be useful in understanding emergence and spread of virulent *C. hominis* subtypes.

## Materials and Methods

### Specimens, Species, and Subtype Determination

Fecal specimens microscopically positive for *Cryptosporidium* spp. were collected during a longitudinal cohort study of enteric diseases in children living in a periurban shantytown in Lima, Peru, during 2004–2006. The field study was conducted in the same community used in a previous longitudinal cohort study of enteric pathogens and had a similar study design (*12*), except that children enrolled in this study were older (mean age 3.43 years vs. 14 days). The study was approved by the institutional review boards of Johns Hopkins University and the Centers for Diseases Control and Prevention.

DNA was extracted from 200 μL of microscopy-positive specimens by using the FastDNA SPIN Kit for Soil (MP Biomedicals, Irvine, CA, USA). *Cryptosporidium* spp. in specimens were genetically characterized at the species level by PCR–restriction fragment length polymorphism analysis of the small subunit rRNA gene (*14*) and at the subtype level by DNA sequence analysis of the gp60 gene (*15*). A total of 53 *C*. *hominis–*positive specimens (1 specimen/child) belonging to 4 gp60 subtype groups were selected for MLST analysis: Ia (9 specimens of IaA13R8 and 1 specimen of IaA13R7), Ib (26 specimens of IbA10G2), Id (6 specimens of IdA10 and 5 specimens of IdA20), and Ie (6 specimens of IeA11G3T3). No specimens used in the study had mixed *C. hominis* subtypes.

### MLST Markers

Among the 8 chromosomes of *Cryptosporidium* spp., chromosome 6 was fully sequenced for 2 *C. parvum* specimens and 1 *C. hominis* specimen, which facilitated search for additional microsatellite and minisatellite markers for population genetics analysis. The 8 polymorphic markers on chromosome 6 (gp60, CP47, CP56, MSC6–5, MSC6–7, TSP8, Mucin1, and DZ-HRGP) used in several MLST and population genetic analyses of *C. hominis* and *C. parvum* (*16*–*18*) were included in this study. We searched for additional microsatellite and minisatellite markers in the chromosome 6 genome by using Tandem Repeats Finder software (http://tandem.bu.edu/trf/trf.html). Of 325 short tandem repeat sequences identified, 46 loci were initially selected at spaced intervals that covered the entire chromosome 6. A nested PCR approach was used for amplification of all potential targets. After an initial evaluation of primers by PCR and DNA sequencing of IaA15R3, IaA20R3, IbA10G2, IdA10, and IeA11G3T3 subtypes of *C. hominis* from the United States and Peru, 24 polymorphic loci (Table 1, Appendix), and the 8 markers previously identified, were used in this study.

**Table 1:** Primer sequences of 24 additional microsatellite and minisatellite loci used in the multilocus typing analysis of Cryptosporidium hominis*

No.	Locus	Polymorphism	Primer	Sequence, 5′→3′	PCR product , bp
1	C6–60	Microsatellite	F1	TGGACAAGAGTGTCGCTTCTA	≈866
		CAC	R1	GTATTGGGTCCTTTCAGCTCA	
			F2	TCCAAAGTACACTGCGTAAATAT	
			R2	TCATGTCTCAACATGCTGAGTA	
2	C6–160	Microsatellite	F1	GTACTCAAGTTCCAGTTGTGGA	≈582
		TA	R1	CCAATACATTCAGAAGACAGTCT	
			F2	CATCCTCAGAAGAGTACTGTA	
			R2	GTGCTGAATGGCTTCCTATTA	
3	C6–190	Microsatellite-	F1	CTATCTCTCAAGCAATGCAAAC	≈534
		AAT	R1	CAAGCTCCATCAGATTATCAATA	
			F2	CAAGTACTGGAATAGTTACTCA	
			R2	CTGGCAATACGTAATGCCTTT	
4	C6–230	Microsatellite	F1	GGCCACTTCATGGTAATACTC	≈627
		TAA	R1	GTCAACGAATCATCCAGAGATT	
			F2	GGTACAATTGGAGGTATCAGT	
			R2	GAATCTCGAACCTGAAGACTT	
5	C6–280	Microsatellite	F1	GATTAAAATCAGGCGGGCCAA	≈764
		TCA	R1	GAGCAATTTCTTGAATAATATTGAG	
			F2	GAGGACTGTGGGGGAATATAA	
			R2	GACAGGAAATCCAATCAATGAATTT	
6	C6–350	Microsatellite	F1	GTTCTTTCAATTGTGTACCATGA	≈715
		ATT	R1	GCTATTGGTTCAAATAATGGTATGA	
			F2	GCATCGCAAATCATCATCTATTT	
			R2	CTACATGGGATTGTTTATATGATA	
7	C6–500′	Microsatellite	F1	GACAAAGAGTATTAGCATCGACA	≈484
		ATA	R1	CTTCATGCTCTAGTTCTTCTAAT	
			F2	GAGTCATATGAAACAGAATCTTT	
			R2	CAAATTGATCGAGATTTGTTGAA	
8	C6–580	Microsatellite	F1	CTGGATGACCAAGGTGTTAAT	≈632
		TCT	R1	CTGTATTGTTACTGTCAATGCTT	
			F2	GAGATGAATATTGAGAATGAATCCA	
			R2	CTCGAGATGATCGATGAATCAT	
9	C6–740	Microsatellite	F1	GAAACAGTTAAAGATAGCTTGGA	≈703
		AT	R1	GAAGATGATGGAATTCTTCTCAT	
			F2	GTATCTTATGTGTATTCAAGCAT	
			R2	CTAATTCTTTGTATGACAAGATCAT	
10	C6–830′	Microsatellite	F1	GAGGCCTTAAAGCTTCTTTTA	≈679
		ATT	R1	GCACTACAACAAGTGGTACTA	
			F2	GAGATTGTATTCTTCTATACCACT	
			R2	CAAGTTCCAATGATAGGATCAGT	
11	C6–830	Microsatellite	F1	GATGTATTACTACCAGAATTGAGA	≈692
		TAG	R1	CTACTCAATCAATGGAAGTTACT	
			F2	CCAACTGATGATATTCCAGTATT	
			R2	GGTATTATTACATCTAGTGGTGA	
12	C6–870	Microsatellite	F1	CAGTGACCGAATTTACTCTCT	≈801
		AT	R1	GTCTCTTTAGTCAAGTTTTCCT	
			F2	CGCTCTTCCACGTACAATTT	
			R2	CGAGAATAAAGCTGACATTTCTT	
13	C6–1000′	Minisatellite	F1	GGAGCCTAATTGTGCTCATAT	≈583
		GATAAAAAG	R1	GGTTATCATGCACTGACTGTA	
		AAGAGGGA	F2	CTCAATTGCTAAAGATGCAGAT	
			R2	GAATCCTGTTGTTCTCCATCT	
14	C6–1420	Minisatellite	F1	GCATTAGAGCATCCATGGTTA	≈534
		GAAAGAGA	R1	CCTGTGGTCTCGATATTCATT	
		GCGT	F2	CATCTTCAGCCGTTGATGAAA	
			R2	GTAAACTCCTTGGAGGTGAAT	
15	C6–2600	Microsatellite	F1	GAGGATGAACTTGTACTGCAAT	≈806
		GCAGCT	R1	CCTAGTATTGGTGGTACTTGTA	
			F2	GGATCGAATTCTGGGTTGAAT	
			R2	GTATCAACTTGCCCTGGAATA	
16	C6–2970	Microsatellite	F1	GACTGTATGCCTTTGTTCCTT	≈723
		GA	R1	GTTGAAGATACTCCTGAAGCT	
			F2	GAGCGTCTTGTCTACTGACTT	
			R2	CTGTCCTCCAGGCATTATTGA	
17	C6–3110′	Minisatellite	F1	GTGGATAAGAGAACCCGTCAT	≈725
		TTCCTCCTA	R1	CACAAATTCTGGTGGGAATAGT	
			F2	CCAGTGATAGTTAATCCAGCT	
			R2	GAGCCAGAAGTCAGTATTTCT	
18	C6–3520	Microsatellite	F1	GTTGGAGTATGACAATTACCTAA	≈748
		TTA	R1	CATCATGGAAATCTTACAGGATT	
			F2	GCATTGTACAACATCCATGTCT	
			R2	GGTGAACATTTTGGATTAGTATCT	
19	C6–3520′	Microsatellite	F1	CTCTGTACAGCTGGTGTAATT	≈577
		TAT	R1	CCTGGTGTGAATCAATGTTGT	
			F2	CTTCTGGAAGCTCACCAAGAA	
			R2	GCTCCTTATAGATTAGCTGAGT	
20	C6–3690	Microsatellite	F1	CTTCTGGAGATTCCATATCATTGA	≈640
		TCT	R1	CCTTAAGGCTGGTTGATATGATT	
			F2	GTGCAAGTGATATTTCCACTATCT	
			R2	CTGTCGAATCACTAGGCAGAA	
21	C6–4110	Microsatellite	F1	CGGAAGATAATGCTCAACTGT	≈709
		ACA	R1	CTGCCTTACTATCATTCGCAT	
			F2	CAAGTGGAAACATTGAGCCAA	
			R2	GGAACTGACTGGTTTGATGAT	
22	C6–5110′	Microsatellite	F1	CACTCTTAATTCCTTCATGGCT	≈533
		CTC	R1	GGTCAATAGGACGTATTTCTTA	
			F2	GCATCCTCAGCTTTAGAATTCT	
			R2	GGTACAGAAGATCAGCTTTCT	
23	C6–5120	Microsatellite	F1	GGATTGATCAGTGACAGTGAA	≈569
		AT	R1	CATAATTGGAGGACTATGTCAAAT	
			F2	GCTGTAGTTCTAAGAGTCCAT	
			R2	GTCTCAAATATCATTAGAACAAGGT	
24	C6–5410	Microsatellite	F1	GACTCAGTTCGAGAGAAGTCA	≈646
		GTAGTG	R1	GGTGAATCATCTTCATGCAGT	
			F2	CATCGAGCGTGCACTTAATCA	
			R2	CTCAGACTCAAGGACCTGAAT	

*All annealing temperatures for primers were 55°C. F, forward; R, reverse.

### MLST PCR and Sequencing

Conditions for nested PCR analysis of each marker were similar to those described (*16*–*18*), except for differences in annealing temperatures specified in Table 1. One negative control and 1 positive control (*C. parvum* IIaA16G2R1 subtype) were used in each PCR. PCR products of the expected size were sequenced in both directions by using an ABI 3130 Genetic Analyzer (Applied Biosystems, Foster City, CA, USA). Sequences obtained from each locus were edited and aligned by using BioEdit 7.04 version (www.mbio.ncsu.edu/BioEdit/bioedit.html) and ClustalX version 1.81 (www.clustal.org/), respectively. Nucleotide sequences of all haplotypes of polymorphic loci were deposited in GenBank under accession nos. JX088398–JX088417 and JX088427–JX088501.

### Analyses of Sequence Polymorphism

For each marker, sequences of 53 *C. hominis* specimens were used for calculation of haplotype diversity (Hd), number of haplotypes, number of polymorphic and segregating sites, intragenic linkage disequilibrium (LD), and intragenic recombination rates by using DnaSP version 5.10.00 (www.ub.es/dnasp/). The ratio of nonsynonymous substitutions per nonsynonymous site to synonymous substitutions per synonymous site was calculated for gp60 and several nearby loci (C6–830′, C6–1000′, C6–1420, CP47, and C6–2600) by using DnaSP. Thereafter, sequences of polymorphic loci were concatenated as a single multilocus contig to calculate gene diversity, interlocus LD, and recombination rates by using DnaSP. Pairwise intergenic LD was also evaluated among polymorphic loci by using the Fisher exact test and Markov chain parameters in Arlequin version 3.1 (http://cmpg.unibe.ch/software/arlequin3/).

### Substructure Analysis

Population substructures were analyzed by using the Bayesian analysis tool STRUCTURE version 2.2 (http://pritch.bsd.uchicago.edu/structure.html). Conversion of microsatellite and minisatellite sequences to allelic data was performed by using the Excel Microsatellite Tool Kit 3.1.1 (http://animalgenomics.ucd.ie/sdepark/ms-toolkit/) and Genepop version 4.0 (http://genepop.curtin.edu.au/). Several analyses of allelic data were performed by using K (likely populations) ranging from 2 to 8 and 50,000 iterations after a burn-in of 50,000 iterations. Output at K = 2–5 provided the best fit to MLST data and were used in further analyses. To provide an alternative view of substructuring, a median-joining algorithm analysis (*19*) was conducted by using Network version 4.6.1.0 (www.fluxus-ngineering.com/sharenet.htm). Each gp60 subtype was further analyzed for LD and recombination by using DnaSP and Arlequin. Population differentiation (*F*_ST_) between IbA10G2 and non-IbA10G2 subtypes was also assessed at each genetic locus by using Arlequin.

## Results

### Multilocus Gene Diversity

Sequence polymorphism among the 53 *C. hominis* specimens was detected at 25 loci (Table 2), including variations in copy numbers of microsatellite or minisatellite repeat and single nucleotide substitutions (SNPs) and insertion and deletion (indels) in the nonrepeat regions. The remaining 7 loci (C6–190, C6–500′, TSP8, C6–830, C6–870, MSC6–5, and Mucin1) were monomorphic in the study population. The Hd of individual polymorphic locus ranged from 0.0377 to 0.9231, and most loci with higher Hd were situated in the first 25% of chromosome 6 (Table 2). Number of haplotypes per locus ranged from 2 to 21, and more haplotypes were at loci C6–60 (21), CP47 (6), gp60 (6), C6–230 (6), and C6–740 (5). MLST analysis confirmed the absence of concurrence of mixed *C. hominis* subtypes in the 53 specimens.

**Table 2:** Intragenic analyses of 53 *Cryptosporidium hominis* specimens at 25 polymorphic loci in chromosome 6*

No.	Locus	Position in chromosome 6	Haplotype diversity	No. haplotypes	Polymorphic sites, bp†	Segregating sites, bp‡	Intragenic LD	Intragenic recombination events
1	CP56	5451–6129	0.4819	3	2	1	NA	NA
2	MSC6–7	12415–12945	0.4819	3	40	1	NA	NA
3	C6–60	22017–22807	0.9231	21	98	3	Y = 1.0000 + 0.0000X	0
4	C6–160	44235–44783	0.6727	3	7	0	NA	NA
5	C6–230	58658–59234	0.4906	6	79	1	NA	NA
6	C6–280	70850–71454	0.5530	3	42	3	Y = 1.0000 + 0.0000X	0
7	C6–350	84688–85277	0.4136	2	21	12	Y = 1.0000 + 0.0000X	0
8	C6–580	138267–138866	0.2409	3	2	2	NA	NA
9	C6–740	188916–189541	0.5791	5	7	1	NA	NA
10	C6–830′	210232–210884	0.1742	2	1	1	NA	NA
11	C6–1000′	251594–252087	0.3832	3	35	20	Y = 1.0000 + 0.0000X	0
12	gp60	266540–267350	0.7090	6	431	297	Y = 0.9729 + 0.0130X	26
13	C6–1420	338725–339242	0.2083	3	13	1	NA	NA
14	CP47	372535–372907	0.8084	6	113	7	Y = 1.0000 + 0.0000X	0
15	C6–2600	606026–606798	0.3570	2	1	1	NA	NA
16	C6–2970	685852–686541	0.3570	2	6	2	NA	NA
17	C6–3110′	721467–722169	0.0377	2	9	0	NA	NA
18	C6–3520	820500–821215	0.4136	2	6	0	NA	NA
19	C6–3520′	822524–823076	0.0377	2	1	1	NA	NA
20	C6–3690	864809–865416	0.4688	2	1	1	NA	NA
21	DZ-HRGP	917600–918139	0.1742	2	3	0	NA	NA
22	C6–4110	956461–957083	0.4115	4	27	5	Y = 1.0000 + 0.0000X	0
23	C6–5110′	1206378–1206774	0.2663	3	8	2	NA	NA
24	C6–5120	1213134–1213677	0.2612	2	6	0	NA	NA
25	C6–5410	1285377–1286003	0.5218	3	9	0	NA	NA

*LD, linkage disequilibrium, where Y is the LD value, and X is the nucleotide distance in kilobases; NA, analysis is not applicable for the locus, which has <3 segregating sites. †Polymorphic sites including insertions/deletions (indels). ‡Segregating sites excluding indels.

Alignment of combined multilocus sequences covered 15,717 bp and 968 polymorphic sites, including 362 segregating sites and 606 indels. Multilocus sequences had 43 multilocus genotypes (MLGs), an Hd of 0.9898, and a nucleotide diversity of 0.339. The frequency of MLGs ranged from 7.5% (1 MLG with 4 specimens), 5.7% (1 MLG with 3 specimens), and 3.8% (5 MLGs each with 2 specimens) to 1.9% (36 MLGs each with 1 specimen). Because 431/968 polymorphic sites and 297/362 segregating sites occurred within the gp60 locus, a second analysis was performed by using concatenated sequences excluding gp60 (14,768 bp) with 537 polymorphic sites, including 65 segregating sites and 472 indel sites. A lower nucleotide diversity of 0.289 was observed. However, the number of MLGs (43) and haplotype diversity (0.9898) remained the same.

### LD and Recombination

Intragenic LD between pairs of segregating sites was assessed for each polymorphic locus. The analysis was possible only for 7 loci (C6–60, C6–280, C6–350, C6–1000′, gp60, CP47, and C6–4110) that had at ≥3 segregating sites. Incomplete intragenic LD (|D′| Y = 0.9729 + 0.0130X), where Y is the LD value and X is the nucleotide distance in kb, was observed at the gp60 locus, and complete intragenic LD (|D′| Y = 1.0000 + 0.0000X) was found at each of the 6 remaining loci (Table 2). An intragenic recombination test identified 26 potential recombination events (Rms) at the gp60 locus and no recombination at the remaining loci (Table 2).

On the basis of the concatenated multilocus sequence data, interlocus LD was assessed over all segregating sites by using pairwise comparisons (*20*). Analysis of combined multilocus sequences of all loci resulted in an overall interlocus genetic association (Z_n_*s*) value of 0.2711 (95% CI 0.0695–0.3918; the probability *p* for expected Z_n_*s* ≤0.2711 was 0.883). Of 47,895 pairwise comparisons, 25,904 were significant by Fisher exact test, and 8,714 were significant after Bonferroni correction (Table 3). In an additional LD analysis per site, strong but incomplete LD (|D′| Y = 0.9289 – 0.0299X) was detected with a negative slope, indicating a decrease in linkage with increased nucleotide distance (Table 3). When analysis was performed after excluding gp60, the test produced a Z_n_*s* value of 0.2184 (95% CI 0.0654–0.4469, p = 0.759). Of 2,080 pairwise comparisons, 921 were significant by Fisher exact test, and 333 were significant after Bonferroni correction. Incomplete LD (|D′| Y = 0.7886 + 0.0051X) was also observed in LD analysis per site (Table 3). Thus, recombination might occur because of incomplete LD. An overall recombination test showed a minimum of 33 potential recombination events and an estimated 5.0 R/gene (Table 3). When analysis was performed after exclusion of gp60, only 6 recombination events and 1.8 R/gene were observed (Table 3). Thus, recombination was occurring mostly at the gp60 locus.

**Table 3:** Pairwise interlocus linkage disequilibrium and recombination analysis of concatenated multilocus sequences from various subtypes of Cryptosporidium hominis*

Population	No. samples	No. segregating sites analyzed	No. pairwise comparisons	No. significant pairwise comparisons†	Zn*s*	|D'|	LD	Estimate of R/gene	Minimum no. recombination events
Including gp60‡									
All	53	362	47,895	25,904 (8,714)	0.2711	Y = 0.9289 – 0.0299X	Incomplete	5.0	33
IaA13R8(7)	10	20	190	0	0.7581	Y = 1.0000 + 0.0000X	Complete	0.001	0
IbA10G2	26	31	465	160 (140)	0.3660	Y = 0.9342 + 0.0001X	Incomplete	0.3	4
IdA10	6	14	91	0	1.0000	Y = 1.0000 + 0.0000X	Complete	0.001	0
IdA20	5	3	3	0	0.3750	Y = 1.0000 + 0.0000X	Complete	0.001	0
IeA11G3T3	6	19	171	0	1.0000	Y = 1.0000 + 0.0000X	Complete	0.001	0
Excluding gp60§									
All	53	65	2080	921 (333)	0.2184	Y = 0.7886 + 0.0051X	Incomplete	1.8	6
IaA13R8(7)	10	20	190	0	0.7581	Y = 1.0000 + 0.0000X	Complete	0.001	0
IbA10G2	26	31	465	160 (140)	0.3660	Y = 0.9302 + 0.0010X	Incomplete	0.3	4
IdA10	6	14	91	0	1.0000	Y = 1.0000 + 0.0000X	Complete	0.001	0
IdA20	5	3	3	0	0.3750	Y = 1.0000 + 0.0000X	Complete	0.001	0
IeA11G3T3	6	19	171	0	1.0000	Y = 1.0000 + 0.0000X	Complete	0.001	0

*Zns, interlocus genetic association; |D'|, linkage disequilibrium (LD) value, where Y is LD value and X is nucleotide distance in kilobases; gp 60, 60-kDa glycoprotein gene. †By Fisher exact test (after Bonferroni correction). ‡Based on concatenated multilocus gene sequence of all loci (15,717 bp).  §Based on concatenated multilocus gene sequence of all loci excluding gp60 (14,768 bp).

### Substructure in *C. hominis*

**Figure 1:**
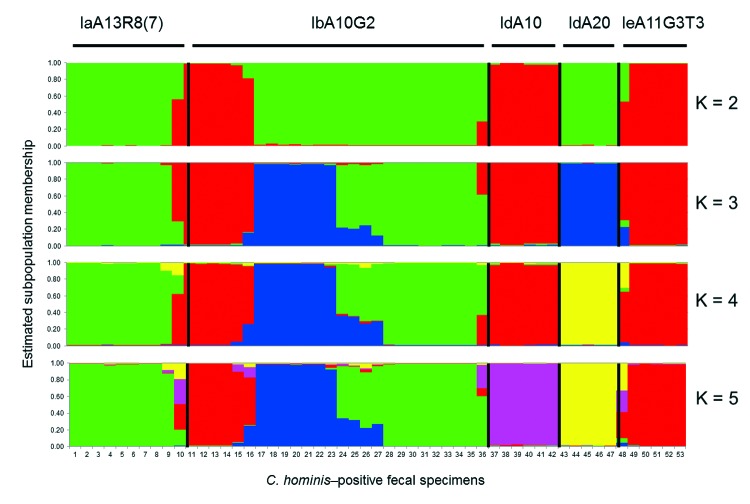
Relationship among various 60-kDa glycoprotein gene subtypes of *Cryptosporidium hominis* by substructure analysis. Predicted population numbers K = 2–5 were applied in STRUCTURE version 2.2 (http://pritch.bsd.uchicago.edu/structure.html) analysis of the data. Colored regions indicate major ancestral contributions. Mixed genotypes are indicated by the pattern of color combinations. Values along the baseline indicate *C. hominis*–positive fecal specimens.

The evolutional relationship among gp60 subtypes of *C. hominis* was inferred by using STUCTURE and predicted population numbers K = 2–5. The ancestral population size K = 5 was considered the best estimate of current population substructure (Figure 1). The most dominant feature in the output was heterogeneity of specimens belonging to subtype IbA10G2 compared with other subtypes. Within 26 specimens of IbA10G2, the pattern of combinations in STRUCTURE suggested a mixture of ancestral types, reflecting likely genetic recombination in the subtype. In contrast, the 10 specimens of the Ia subtype (including 9 specimens of IaA13R8 and 1 specimen of IaA13R7) had mostly a single pattern, thus providing strong support for it being a separate population with a unique ancestry. Likewise, intrasubtype homogeneity was also observed in 6 specimens of IdA10, five specimens of IdA20, and 6 specimens of IeA11G3T3. Within the Id subtype, specimens of IdA10 and IdA20 showed different patterns regardless of the K value, indicating that these 2 Id subtypes had distinct ancestries with little mixture and genetic recombination. On the basis of patterns at K = 5 (Figure 1), some IbA10G2 specimens had a mixture of patterns, which were the dominant patterns in subtype IaA13R8 and subtype IeA11G3T3, respectively, suggesting that these IbA10G2 specimens might have resulted from genetic exchange between the ancestor of subtype IbA10G2 and the ancestor of subtype IaA13R8 or IeA11G3T3.

**Figure 2:**
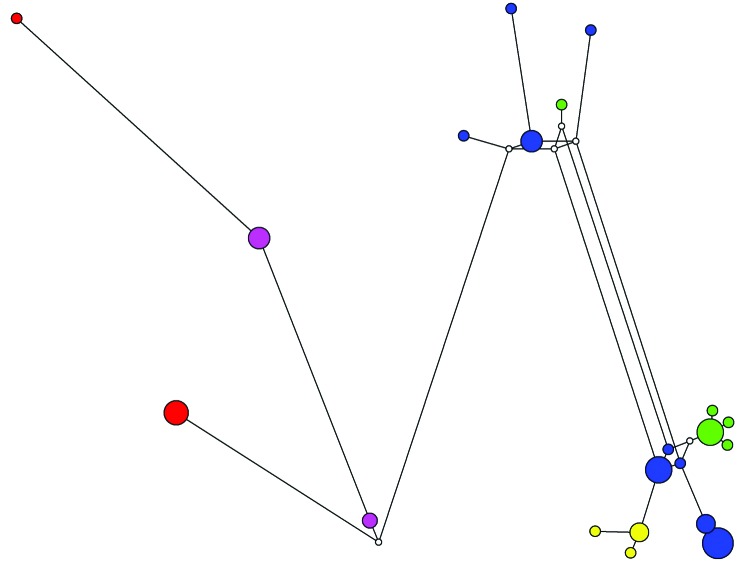
Median-joining network for various subtypes of *Cryptosporidium hominis*. Circles are proportional to the frequency of each multilocus genotype (MLG) (21 MLGs on the basis of segregating sites of concatenated sequences excluding the 60-kDa glycoprotein gene). The color of each circle represents the 60-kDa glycoprotein gene subtypes of the *C. hominis* specimens: IaA13R8 (green), IbA10G2 (blue), IdA10 (purple), IdA20 (yellow), and IeA11G3T3 (red). Length of lines connecting MLGs is proportional to the number of single-nucleotide polymorphisms.

We also conducted a median-joining network analysis of the MLST data for the 53 specimens. Among 21 MLGs generated on the basis of segregating sites of concatenated sequences, excluding gp60, we did not find any shared MLGs between gp60 subtypes of *C. hominis* (Figure 2). Compared with STRUCTURE analyses, the results of network analysis showed similarity to patterns at K = 2 (Figure 1), and supported the conclusion on the heterogeneity of subtype IbA10G2. In network analysis, central types are usually possible ancestors, and peripheral types are descendants (*21*). Because we did not find any MLG as the central type, it was impossible to define a single ancestral line that gave rise to other lines. This finding could be caused by the small sample size and few MLGs in this study.

### Comparative Population Genetics of IbA10G2

**Figure 3:**
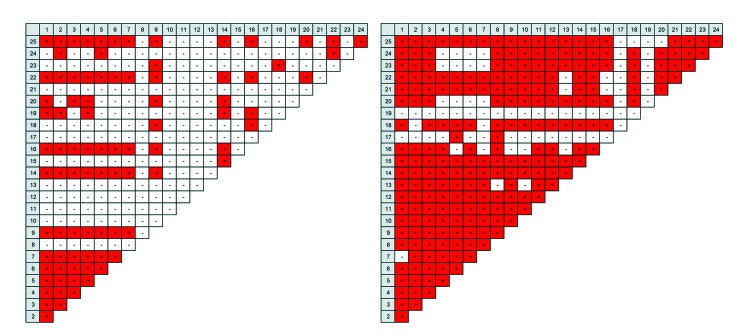
Pairwise intergenic linkage disequilibrium (LD) matrix among 25 polymorphic loci of *Cryptosporidium hominis*. The 25 polymorphic loci are indicated in gray (see Table 2 for identification of loci). Significant LD between loci is indicated in red. Subtype IbA10G2 (A), which has 87 instances of pairwise LD, has fewer LD in comparison with the remaining subtypes (B), which have 227 instances of pairwise LD. In IbA10G2, loci around the 60-kDa glycoprotein gene (locus 12) have no LD with any other loci in chromosome 6.

The population genetics of various gp60 subtypes was assessed by analyses of LD and recombination rates. In pairwise interlocus LD analysis of multilocus sequences including or excluding gp60, strong but incomplete LD (|D′| Y = 0.9342 + 0.0001X or |D′| Y = 0.9302 + 0.0010X) was observed in subtype IbA10G2, suggesting recombination within this subtype. In contrast, all other subtypes showed complete LD (|D′| Y = 1.0000 + 0.0000X) among all sites, indicative of no genetic recombination within these subtypes (Table 3). In addition, pairwise intergenic LD was also evaluated between 25 loci, resulting in 87 instances of significant pairwise LD (p<0.05) observed in subtype IbA10G2 compared with 227 significant LD in the remaining subtypes (Figure 3). In IbA10G2 specimens, loci around gp60 (locus 12) had no LD with any other loci in chromosome 6. This finding indicated genetic recombination in subtype IbA10G2 at loci near gp60.

To validate these observations, recombination tests were conducted for all gp60 subtypes. Using full multilocus sequences, we found that among 33 potential Rms observed in overall recombination analysis, 4 Rms were detected in subtype IbA10G2 but no Rm was found within each of the remaining subtypes (Table 3). When analysis was performed that excluded gp60, among 6 potential Rms in the overall recombination analysis, 4 Rms were still detected in subtype IbA10G2 but no Rm was seen in the other subtypes (Table 3). Thus, genetic recombination occurred only within the subtype IbA10G2, a conclusion in agreement with results of the STRUCTURE and LD analyses.

**Figure 4:**
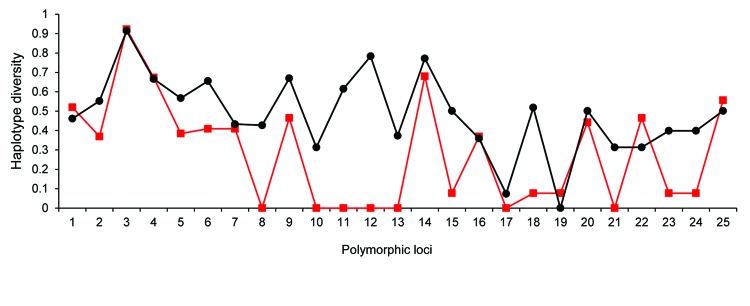
Genetic variation in haplotype diversity at 25 polymorphic loci in chromosome 6 of *Cryptosporidium hominis* (see Table 2 for identification of loci). Red squares indicate subtype IbA10G2 and black circles indicate non-IbA10G2 subtypes. A homogeneity (reduced haplotype diversity) of subtype IbA10G2 was seen in 4 loci around the 60-kDa glycoprotein gene.

The genetic determinant for differences between IbA10G2 and non-IbA10G2 subtypes was assessed by comparison of haplotype diversity of the 2 groups at all 25 polymorphic loci in chromosome 6. At most of the genetic loci, the haplotype diversity of the 2 groups was similar, and there was no clear population differentiation at these loci (Figure 4, Table 4). However, at 4 loci (C6–830′, C6–1000′, gp60, C6–1420) around gp60, an absence of genetic diversity was observed in subtype IbA10G2 compared with non-IbA10G2 subtypes (Figure 4). The region (129 kb) of homogeneity in subtype IbA10G2 was located in a 210–339-kb region in chromosome 6. Pairwise *F*_ST_ analysis showed highly significant differentiation between the IbA10G2 subtype and non-IbA10G2 subtypes at 4 loci around gp60, C6–1000′ (p<0.00001), gp60 (p<0.00001), CP47 (p<0.00001), and C6–2600 (p<0.001). The only other locus that showed such a level of differentiation between the 2 groups was C6–3520 (Table 4).

**Table 4:** Estimates of haplotype diversity in IbA10G2subtype and non-IbA10G2 subtypes of *Cryptosporidium hominis* and comparison of 2 populations at each locus by pairwise *F*_ST_ analysis*

No.	Locus	Haplotype diversity		Population pairwise *F*_ST_	p value ± SEM
		IbA10G2 subtype	Non-IbA10G2 subtypes		
1	CP56	0.5200	0.4615	−0.03454	0.88867 ± 0.0104
2	MSC6–7	0.3692	0.5527	0.07491	0.10156 ± 0.0074
3	C6–60	0.9231	0.9145	0.00922	0.21387 ± 0.0105
4	C6–160	0.6738	0.6667	0.00735	0.31738 ± 0.0131
5	C6–230	0.3846	0.5670	0.05070	0.07422 ± 0.0069
6	C6–280	0.4092	0.6553	0.06300	0.06836 ± 0.0076
7	C6–350	0.4092	0.4330	−0.03736	0.99902 ± 0.0002
8	C6–580	0.0000	0.4274	0.17205	0.00781 ± 0.0028†
9	C6–740	0.4646	0.6695	0.03343	0.12891 ± 0.0126
10	C6–830′	0.0000	0.3134	0.15031	0.04883 ± 0.0067‡
11	C6–1000′	0.0000	0.6154	0.30288	0.00000 ± 0.0000§
12	gp60	0.0000	0.7835	0.60340	0.00000 ± 0.0000§
13	C6–1420	0.0000	0.3732	0.15665	0.02051 ± 0.0038‡
14	CP47	0.6800	0.7721	0.18033	0.00000 ± 0.0000§
15	C6–2600	0.0769	0.5014	0.29888	0.00098 ± 0.0010§
16	C6–2970	0.3692	0.3590	−0.03901	0.99902 ± 0.0002
17	C6–3110’	0.0000	0.0741	−0.00143	0.99902 ± 0.0002
18	C6–3520	0.0769	0.5185	0.42036	0.00000 ± 0.0000§
19	C6–3520’	0.0769	0.0000	0.00148	0.48535 ± 0.0148
20	C6–3690	0.4431	0.5014	−0.01700	0.55859 ± 0.0145
21	DZ-HRGP	0.0000	0.3134	0.15031	0.03809 ± 0.0056‡
22	C6–4110	0.4646	0.3134	0.10817	0.01855 ± 0.0048‡
23	C6–5110’	0.0769	0.3989	0.17072	0.00781 ± 0.0028†
24	C6–5120	0.0769	0.3989	0.14126	0.05664 ± 0.0066
25	C6–5410	0.5569	0.5014	−0.02614	0.67383 ± 0.0182

*F_ST_, population differentiation. †p<0.01. ‡p<0.05. §p<0.001.

## Discussion

Like other apicomplexan parasites, the life cycle of *Cryptosporidium* spp. has a sexual phase, during which sexual recombination can occur between genetically distinct strains (*22*). *C. parvum*, the species that infects humans and some animals, undergoes meiotic recombination between different lineages in genetic-crossing experiments (*23*,*24*). LD analyses of natural *C. parvum* populations have also shown genetic recombination in most study areas (*22*,*25*–*28*). In contrast, *C. hominis* is primarily infectious to humans, and previous MLST studies showed a clonal population structure. Genetic recombination was believed to be rare or nonexistent in *C. hominis* (*17*,*25*,*28*–*30*). However, the small number of markers used in previous studies might have resulted in relatively low resolution in population structure analysis, which could have led to failure in detecting genetic recombination in *C. hominis*. In the present study, we examined population substructure of *C. hominis* in a cohort of children living in a small study area by using 32 genetic markers.

On the basis of multilocus sequence data and allelic profiles for 53 specimens, analysis showed strong LD among 25 polymorphic loci, suggesting an overall nonpanmictic population structure of *C. hominis*. A recombination test showed only limited genetic recombination at the gp60 locus. Thus, the high level of LD and limited recombination found in the overall population could be explained by an essential clonal population structure of *C. hominis* in the field site in Peru, which is consistent with information on *C. hominis* population genetics in other countries (*17*,*25*,*28*,*29*).

Results of LD and recombination analyses suggest that limited recombination in the study population of *C. hominis* occurred mostly at gp60 or loci around gp60. Thus far, the gp60 gene is the most polymorphic marker identified in the *Cryptosporidium* genome. Because of its high sequence heterogeneity, gp60 has become the most widely used gene in *Cryptosporidium* spp. subtyping, which categorizes *C. hominis* and *C. parvum* into several subtype groups and various subtypes within each subtype group (*2*). The gp60 gene encodes glycoproteins gp15 and gp45, which are implicated in attachment to and invasion of host cells (*31*,*32*). Because attachment of sporozoites to epithelial cells and invasion of the host cell membrane are crucial steps in the pathogenesis of cryptosporidiosis, these 2 glycoproteins are presumed to be surface-associated virulence determinants that may be under host immune selection, which might explain the extensive polymorphism in the gp60 gene (*27*). Genetic recombination appears to be associated with high sequence polymorphism in the gp60 gene (*27*). However, a less speculative understanding of the role the gp60 gene in pathogenesis of cryptosporidiosis requires further investigations.

Recent studies have suggested that telomeric/subtelomeric regions are highly polymorphic and might encode putative virulence factors (*33*,*34*). However, these studies did not compare phenotypic differences among isolates, and data for the present study do not relate directly to sequence variations at telomeres. In the present study, because *C. hominis* subtype IbA10G2 was shown to be more virulent than other subtypes in the study community (*12*), we compared population genetics of IbA10G2 and other gp60 subtypes. Heterogeneity within subtype IbA10G2 was observed in STRUCTURE and Network analyses compared with homogeneity within each of the remaining subtypes. This finding suggested a mixture of ancestral genetic elements and genetic recombination in virulent subtype IbA10G2. This finding was confirmed by incomplete LD and several recombination events (4 Rms) found in IbA10G2. In addition, the pattern of shared ancestral types in this study suggests that genetic exchange might have occurred between the ancestor of subtype IbA10G2 and the ancestor of subtype IaA13R8 or IeA11G3T3.

Pairwise *F*_ST_ analysis of 25 polymorphic loci in chromosome 6 between the virulent IbA10G2 subtype and non-IbA10G2 subtypes showed population differentiation at 4 loci around gp60. When a locus shows extraordinary levels of genetic population differentiation compared with other loci, this finding might be interpreted as evidence for positive selection (*35*). Thus, the region around gp60 in subtype IbA10G2 might be under selection pressure. This finding was further confirmed by comparison of haplotype diversity of the 2 groups at the 25 polymorphic loci. Although a similar Hd was found between the 2 groups in most regions of chromosome 6, a region of Hd reduction was observed in subtype IbA10G2 in a 129-kb region flanking gp60, compared with the non-IbA10G2 subtypes.

One explanation for this pattern of genetic diversity is that the region surrounding gp60 was probably affected by selective sweep or genetic hitchhiking caused by selection of the virulence subtype. A hallmark of a selective sweep is a chromosomal region with reduced diversity associated with a specific phenotype. In previous studies of other apicomplexan parasites, similar patterns of reduced genetic variations were observed in chromosomal regions surrounding sites under selection pressure. Wootton et al. (*36*) found a dramatic reduction in genetic variation in chloroquine-resistant parasites within a region spanning >200 kb around the *Plasmodium falciparum* chloroquine-resistance transporter gene in chromosome 7 as the result of selection for chloroquine resistance. Nair et al. (*37*) observed decreased variation in an ≈100-kb region flanking the dihydrofolate reductase gene in chromosome 4 of *P. falciparum* in association with pyrimethamine resistance. Consistent with these findings, we detected reduced sequence variation around gp60 in virulent subtype IbA10G2, suggesting that the 129-kb region surrounding gp60 in chromosome 6, perhaps gp60 itself, might be involved in selection for virulent gp60 subtype IbA10G2 in *C. hominis*. In a comparison of the number of nonsynonymous substitutions per nonsynonymous site to the number of synonymous substitutions per synonymous site analysis of loci around gp60, we confirmed the presence of positive section at least at the CP47 locus.

The reason for exclusive occurrence of genetic recombination in IbA10G2 is not clear. Because IbA10G2 is the major subtype of *C. hominis* responsible for numerous waterborne and foodborne outbreaks of cryptosporidiosis in many countries (*2*), it is transmitted frequently among humans, resulting in increased probability of mixed infections with other genetically heterogeneous subtypes, especially in countries in Europe in which autochthonous *C. hominis* infections are caused mostly by IbA10G2 and imported cases by other subtypes (*38*). In a long evolutionary process, the common occurrence and biologic fitness of the gp60 IbA10G2 subtype may facilitate genetic recombination with other *C. hominis* subtypes and subsequent spread of the recombinant parasite with the fitness gene. Recently, genetic recombination was shown to be a key strategy for selection of virulent clones of *Toxoplasma gondii*, an apicomplexan parasite with a largely clonal population structure in North America and Europe (*39*).

In conclusion, we have shown complex substructures in a natural *C. hominis* population in a cohort of children living in a small community in Peru. Although *C. hominis* from the community has an overall clonal population structure, genetic recombination occurs within subtype IbA10G2 around the gp60 locus, which might be involved in pathogenicity. Common occurrence of its parental subtypes and biologic fitness of the recombinant subtype with the IbA10G2 trait have probably facilitated genetic exchange and spread of the virulent subtype. In addition, we were able to localize selection for the virulent subtype IbA10G2 to a 129-kb region surrounding gp60 in chromosome 6. These observations could improve our understanding of emergence and spread of virulent *C. hominis* subtypes.
